# Gestational diabetes mellitus prevalence in Brazil: a systematic review and meta-analysis

**DOI:** 10.1590/0102-311XEN064919

**Published:** 2024-09-09

**Authors:** Lucas Pitrez Mocellin, Hewellynn de Azeredo Gomes, Lincoln Sona, Gabrielle Maria Giacomini, Eduarda Pires Pizzuti, Gabriéli Borges Nunes, Túlio Marcos Zanchet, Juliana Lopes de Macedo

**Affiliations:** 1 Universidade Federal do Pampa, Uruguaiana, Brasil.; 2 Universidade Federal de Ciências da Saúde de Porto Alegre, Porto Alegre, Brasil.

**Keywords:** Gestational Diabetes Mellitus, Prevalence, Meta-Analysis, Systematic Review, Diabetes Mellitus Gestacional, Prevalência, Metanálise, Revisão Sistemática, Diabetes Mellitus Gestacional, Prevalencia, Metaanálisis, Revisión Sistemática

## Abstract

This study estimates gestational diabetes mellitus prevalence in Brazil. A systematic review was conducted with articles published between 2010 and 2021 on the PubMed, Scopus, Google Scholar, SciELO, LILACS and Virtual Health Library databases, as well as gray literature. Data were extracted using a standardized instrument together with the risk of bias assessment tool proposed by Hoy et al. A meta-analysis with robust variance and random effects was developed. Heterogeneity was verified using I^2^ and publication bias was assessed using funnel plot and Egger’s test. Prevalence according to risk of bias, diagnostic criteria and country’s regions was determined by subgroup analyses. A total of 32 studies were included, representing 21,942 women. gestational diabetes mellitus pooled prevalence was 14% (95%CI: 11.0; 16.0), considerably higher than estimates from previous studies. Regarding risk of bias, studies with low, medium, and high risk showed a pooled prevalence of 12%, 14% and 14%, respectively. Overall GRADE certainty of evidence rating was low. Most studies used the International Association of Diabetes in Pregnancy Study Group (IADPSG) criteria or the adapted IADPSG, showing a pooled prevalence of 15% and 14%, respectively. As for region, the pooled prevalence was higher in the Southeast (14%) and lower in the Central-West (9%). This is the first systematic review to provide evidence on gestational diabetes mellitus prevalence at a national level and to demonstrate considerable heterogeneity among articles and the influence of region, diagnostic criteria and study quality on the referred indicator.

## Resumo

Este artigo estimou a prevalência da diabetes gestacional no Brasil. Foi realizada uma revisão sistemática e metanálise com artigos publicados de 2010 até 2021 nas bases de dados PubMed, Scopus, Google Scholar, SciELO, LILACS e Biblioteca Virtual em Saúde, além de literatura cinzenta. Os dados foram extraídos usando um instrumento padronizado juntamente com o instrumento de avaliação de risco de viés de Hoy et al. Posteriormente, foi desenvolvida uma metanálise com variância robusta e efeitos aleatórios. A heterogeneidade foi verificada pelo uso do I^2^ e o viés de publicação foi avaliado pelo gráfico de funil e pelo teste de Egger. Análises de subgrupos foram realizadas para determinar a prevalência de acordo com o risco de viés, critérios diagnósticos e regiões do país. Ao todo, 32 estudos foram incluídos nesta metanálise, totalizando 21.942 mulheres. A prevalência combinada de diabetes gestacional no Brasil foi de 14% (IC95%: 11,0; 16,0), consideravelmente superior às estimativas de estudos anteriores. Em relação ao risco de viés, estudos com baixo, médio e alto risco mostraram prevalência combinada de 12%, 14% e 14%, respectivamente. Em relação à certeza da evidência (abordagem GRADE), a classificação geral foi baixa. A maioria dos estudos utilizou os critérios do Grupo de Estudo da Associação Internacional de Diabetes na Gravidez (IADPSG) e do IADPSG adaptado, mostrando uma prevalência combinada de 15% e 14%, respectivamente. Em relação às regiões, a prevalência combinada foi maior no Sudeste (14%) e menor no Centro-oeste (9%). Esta foi a primeira revisão sistemática a fornecer evidências sobre a prevalência de diabetes gestacional em nível nacional, demonstrando considerável heterogeneidade entre os artigos e a influência da região, dos critérios diagnósticos e da qualidade dos estudos sobre o referido indicador.

## Resumen

Este artículo estimó la prevalencia de diabetes gestacional en Brasil. Se realizó una revisión sistemática y metaanálisis con artículos publicados del 2010 al 2021 en las bases de datos PubMed, Scopus, Google Scholar, SciELO, LILACS y Biblioteca Virtual en Salud, además de literatura gris. Los datos se extrajeron usando un instrumento estandarizado junto con el instrumento de evaluación del riesgo de sesgo de Hoy et al. Posteriormente, se desarrolló un metaanálisis con varianza robusta y efectos aleatorios. La heterogeneidad se verificó mediante el I^2^, y el sesgo de publicación se evaluó por medio del gráfico en embudo y la prueba de Egger. Se realizaron análisis de subgrupos para determinar la prevalencia según el riesgo de sesgo, criterios diagnóstico y regiones del país. En total, se incluyeron 32 estudios en este metaanálisis, con un total de 21.942 mujeres. La prevalencia combinada de diabetes gestacional en Brasil fue del 14% (IC95%: 11,0; 16,0), considerablemente más alta que las estimaciones de estudios anteriores. Con relación al riesgo de sesgo, los estudios con riesgo bajo, medio y alto mostraron una prevalencia combinada del 12%, del 14% y del 14%, respectivamente. En cuanto a la certeza de la evidencia (enfoque GRADE), la clasificación general fue baja. La mayoría de los estudios utilizó los criterios del Grupo de Estudio de la Asociación Internacional de Diabetes en el Embarazo (IADPSG) y del IADPSG adaptado, lo que muestra una prevalencia combinada del 15% y del 14%, respectivamente. Con relación a las regiones, la prevalencia combinada fue mayor en el Sudeste (14%) y menor en el Centro-Oeste (9%). Esta fue la primera revisión sistemática que proporcionó evidencias sobre la prevalencia de diabetes gestacional en el ámbito nacional, lo que demuestra una considerable heterogeneidad entre los artículos y la influencia de la región, los criterios diagnósticos y la calidad de los estudios sobre este indicador.

## Introduction

Gestational diabetes mellitus consists in the state of hyperglycemia during pregnancy with glycemic levels that indicate no previous diabetes mellitus diagnosis ^1^. Its development involves factors such as a state of insulin resistance and hormonal and metabolic changes caused by the body’s adaptation to the fetal needs, as well as nutritional and genetic factors ^2^. Gestational diabetes mellitus is one of the leading causes of morbidity and mortality in pregnant women and newborns, representing a global public health issue with repercussions for the maternal-fetal binomial ^3^, such as childhood obesity or type 2 diabetes mellitus. Consequently, it results in higher public health expenses which could be avoided with early diagnoses and effective interventions to aid pregnant women during this period ^3^ ^,^ ^4^.

Gestational diabetes mellitus has several diagnostic criteria resulting in a high diversity for its estimated prevalence. The American Diabetes Association (ADA) and the World Health Organization (WHO) recommend adopting the International Association of Diabetes in Pregnancy Study Group (IADPSG) diagnostic criteria for gestational diabetes mellitus diagnosis, confirmed by testing the levels of fasting blood glucose or using oral glucose tolerance test (OGTT) 75g between 24-28 weeks of pregnancy, period in which insulin resistance is significantly increased ^5^. In 2013, WHO extended the IADPSG criterion validity for any gestational age and established OGTT values after 2 hours up to 199mg/dL, thus avoiding convergence with the diabetes mellitus criteria ^1^.

Globally, gestational diabetes mellitus prevalence varies between 0.3% and 28% ^6^. In 2015, approximately 17.8 million deliveries with neonates born alive to pregnant women between 20 and 49 years old were affected by this condition ^7^. In Brazil, prevalence data show considerable variability as shown by a multicenter cohort study with 5,564 pregnant women that estimated a 18% prevalence (95% confidence interval - 95%CI: 16.9; 19.0) ^8^, whereas a cohort research with 4,131 participants observed a 2.6% prevalence (95%CI: 2.1; 3.1) ^9^, despite being based on self-reported answers.

The lack of systematic reviews and meta-analyses on gestational diabetes mellitus in Brazil, as well as the impact of this clinical outcome on national health, justifies an in-depth investigation of observational studies conducted in the country. In this context, this article estimated the gestational diabetes mellitus pooled prevalence in Brazil, a relevant data to subsidize planning and administration of interventions such as public policies, health services and programs aimed at reducing the level of gestational diabetes mellitus impact and improving mother and child health ^10^ ^,^ ^11^. Additionally, it categorized the pooled prevalence evaluation according to country region, the gestational diabetes mellitus diagnostic criteria used and the risk of bias in the analyzed articles.

## Methods

This systematic review and meta-analysis was developed according to the *Cochrane Handbook for Systematic Reviews* guidelines and the *Preferred Reporting Items for Systematic Reviews and Meta Analyses* (PRISMA) precepts. It was registered on the PROSPERO platform (code CRD42022293743).

### Search strategy and databases

Intending to access all eligible studies for inclusion in the data set, we performed a systematic search for articles in the PubMed, Scopus, Google Scholar, SciELO, LILACS and Virtual Health Library databases, as well as the analysis of gray literature researched in annals and works published in Brazilian and Latin-American congresses in the fields of Gynecology and Obstetrics, Endocrinology, and Metabology. For each database a search strategy was developed using sensitive terms to the subject (Supplementary Material - Box S1; https://cadernos.ensp.fiocruz.br/static//arquivo/suppl-e00064919_9189.pdf). Articles in Portuguese, Spanish and English were considered.

### Study eligibility

This systematic review included observational or diagnostic studies on gestational diabetes mellitus that presented data about the prevalence of this disease in Brazil published between 2010 and 2021. The year 2010 represents the milestone of adherence to the IADPSG diagnostic criteria for gestational diabetes mellitus, adopted by WHO and the Brazilian Ministry of Health ^4^. Exclusion criteria consisted of studies that did not address the research question, duplicated studies, qualitative studies, article reviews, case reports, narrative reviews and conference abstracts with incomplete information or that did not answer the investigators, editorials, commentaries, letters to the editor, author responses and other publications that did not include quantitative data.

### Study selection

Studies identified in each database were imported into Microsoft Word (https://products.office.com/). After removing duplicates using the Copyspider tool (https://copyspider.com.br/), the title, abstract and full text of the articles were analyzed based on the established inclusion and exclusion criteria. Pairs independently performed this analysis and, in case of disagreement, a third evaluator was responsible for the final decision.

### Data extraction

Data on authors, title, year of publication, journal, database, language, location, region of the country and state, year of investigation, study design and main objective were extracted from the selected studies using Google Forms (https://docs.google.com/forms). Inclusion and exclusion criteria, size of total and studied samples, lost sample size, diagnostic criteria, gestational period at time of diagnosis, gestational diabetes mellitus prevalence and respective confidence interval and risk factors for the mother and child were also observed. Pairs independently performed the extraction and, in case of disagreement, a third evaluator made the final decision.

### Outcomes and diagnostic criteria

Gestational diabetes mellitus prevalence was obtained by calculating the ratio between the number of pregnant women diagnosed with gestational diabetes mellitus and the total number of pregnant women in the studied sample. Gestational diabetes mellitus diagnostic criteria varied between articles. IADPSG criterion was attributed when the study followed the definition below or cited its use: fasting blood glucose ≥ 92mg/dL and ≤ 125mg/dL at the first prenatal visit or at least one of the OGTT with 75g values of ≥ 92mg/dL in fasting, ≥ 180mg/dL after one hour and ≥ 153mg/dL after two hours, performed between 24 and 28 weeks of gestation ^1^. Adapted IADPSG was considered when the article adopted the specifications above with some alteration in the testing period or blood glucose values. The 2010 ADA criterion was met if gestational diabetes mellitus diagnosis was confirmed with an OGTT 100g value greater than or equal to at least two of the values: 95mg/dL in fasting, 180mg/dL after one hour, 155mg/dL after two hours, and 140mg/dL after three hours ^12^. Studies that made the diagnosis using fasting plasma glucose ≥ 126mg/dL and/or OGTT 75g ≥ 140mg/dL after two hours followed the 1999 WHO guidelines ^1^. The Brazilian Diabetes and Pregnancy Task Force (GTDG, acronym in Portuguese) 2001 criterion corresponds to diagnosis based on fasting glucose ≥ 110mg/dL or OGTT 75g ≥ 140mg/dL after two hours ^13^. Finally, articles that did not inform or that did not specify the adopted diagnostic criteria were listed as not informed and non-accurate criteria, respectively.

### Quality evaluation

Study quality was assessed by analyzing risk of bias based on a tool developed by Hoy et al. ^14^ which has been used in systematic reviews aiming to assess the prevalence of a health problem or event ^10^ ^,^ ^15^ ^,^ ^16^. The instrument consists of ten items that address four different bias domains and an overall summary assessment based on the responses to the previous items. Their topics correspond to external (items 1 to 4, whose domains are selection and non-response bias) and internal (items 5 to 10, whose domains are measurement and analysis) study validity dimensions ^14^. Each article was classified according to the answers to individual items: “yes”, if the item was answered or “no”, if the information was insufficient or not contemplated, resulting in a final classification depending on the added result: 8 or more “yes” answers indicated low risk of bias; 6 to 7 “yes” answers, moderate; and 5 or less “yes” answers a high risk of bias. Similar categorization was used in other systematic review studies ^10^ ^,^ ^16^. Some conventions were adopted to standardize the risk of bias classification. Regarding external validity, study of local population, exclusion criteria selective to a certain population or use of a convenience sample were considered high risk. As for internal validity, information obtained from only one source (e.g., only from medical records), unspecified diagnostic criteria, different data collection between individuals in the sample, unspecified time of diagnosis (gestational week) or no information on the numerator and denominator used to calculate prevalence indicated high risk.

The GRADE (Grades of Recommendation, Assessment, Development, and Evaluation) assessment tool for prognosis studies was used to rate the certainty of the evidence generated ^17^. A summary of findings was developed, explaining the decision regarding the five criteria (risk of bias, inconsistency, imprecision, indirectness and publication bias).

### Data analysis

All eligible studies were included in the systematic review for constructing a database based on the collection instrument. We developed a meta-analysis with robust variance and random effects using the Stata software, version 16 (https://www.stata.com), in which we prepared the forest plot and estimated the summary measure for the pooled prevalence data together with its confidence interval. Heterogeneity between studies was verified by calculating I^2^ variability (low < 25%, moderate 25-50% and high > 50%). Gestational diabetes mellitus pooled prevalence for each country region and according to risk of bias was estimated by subgroup analyses. Pooled prevalence was also analyzed according to the gestational diabetes mellitus diagnostic criteria used. Publication bias was verified by a funnel plot, Egger’s test and trim-and-fill sensibility analysis. Finally, a meta-regression analysis for random effects was performed to verify trends over time considering the years of data collection. The first year was considered when the study presented a data collection longer than one year. Articles lacking this information were excluded from the meta-regression analysis.

## Results

**Figure 1:**
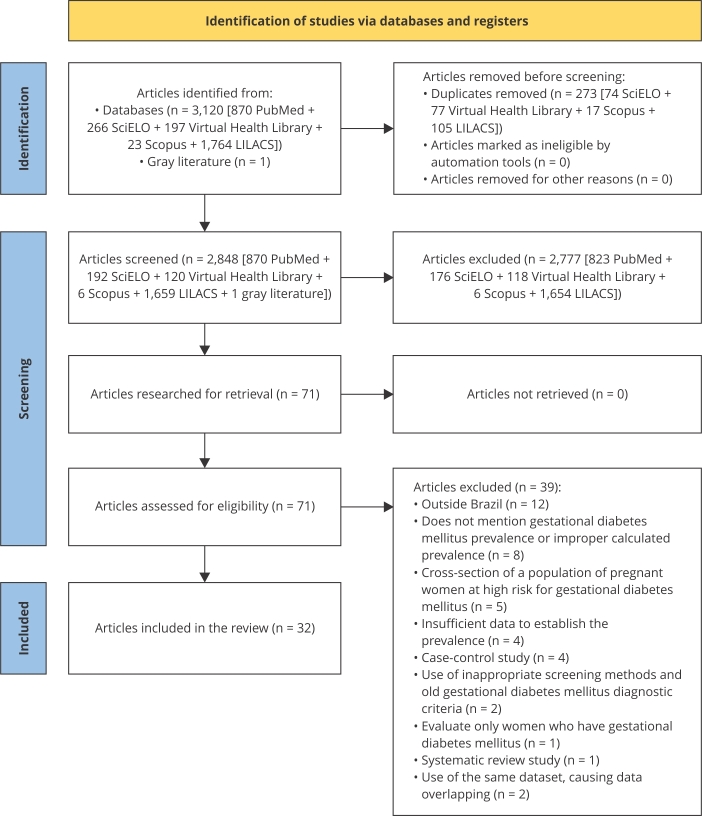
Flowchart of studies included in the systematic review and meta-analysis of gestational diabetes mellitus prevalence in Brazil between 2010 and 2021.

Figure 1 summarizes the selection process for the studies included in this systematic review. A total of 3,121 articles were identified, 3,120 from five different databases and 1 retrieved from gray literature. By reading the titles and abstracts, 273 duplicates were identified resulting in 2,848 studies for screening. Of these, 2,776 articles were excluded in a later evaluation for not addressing the research topic, not presenting prevalence data, and not considering Brazil as the source of analysis. Of the 71 articles pre-selected for reading, 39 were removed for meeting the exclusion criteria thus totaling 32 studies included in the review.

Total sample consisted of 21,942 women from four different Brazilian regions. Seven studies were conducted in the Northeast, two in the Central-West, 11 in the Southeast and ten in the Southern region (Table 1). Regarding study design, 17 were cross-sectional studies, 13 were prospective cohort studies and four were retrospective cohort studies.

**Table 1:** Characteristics of studies included in the systematic review and meta-analysis of gestational diabetes mellitus prevalence in Brazil between 2010 and 2021.

Study (Year)	State/Region	Investigation period	Study design	Full sample	Sample studied	Prevalence (%)	Diagnostic criteria	Gestational age
Pinheiro et al. ^43^ (2018)	Rio Grande do Sul	NI	Prospective cohort	295	219	31.9	IADPSG adapted	NI
do Nascimento et al. ^44^ (2019)	Pernambuco	November 2012/February 2014	Prospective cohort	907	544	17.4	IADPSG	24-28 weeks
Nunes et al. ^45^ (2020) *	Santa Catarina	NI	Retrospective cohort	NI	120	18.3	IADPSG	24-28 weeks
dos Santos et al. ^46^ (2020)	Rio Grande do Sul	January/December 2016	Cross-sectional	3,411	2,313	5.4	IADPSG adapted	NI
Alves et al. ^47^ (2020)	Pernambuco	March 2016/September 2018	Prospective cohort	627	518	16.8	IADPSG adapted	24-28 weeks
Nicolosi et al. ^48^ (2020)	Pernambuco, Ceará, São Paulo, Rio Grande do Sul	July 2015/July 2018	Prospective cohort	1,373	1,008	14.1	IADPSG adapted	NI
Zhao et al. ^49^ (2016)	São Paulo	September 2011/December 2013	Cross-sectional	4,740	354	3.1	WHO 1999	NI
Guttier et al. ^32^ (2019)	Rio Grande do Sul	January/December 2004	Prospective cohort	4,261	3,182	3.3	NI	NI
Sirimarco et al. ^50^ (2017)	São Paulo	January 2008/December 2014	Cross-sectional	482	482	40.2	ADA 2010	NI
Santos et al. ^51^ (2012)	Northeast	May 2007/May 2008	Prospective cohort	204	183	3.4	GTDG 2001	NI
Fagundes et al. ^52^ (2016)	São Paulo	NI	Cross-sectional	58	58	22.4	IADPSG	24-28 weeks
Siqueira et al. ^19^ (2019)	Federal District	June 2013/June 2015	Cross-sectional	519	337	28.2	NI	NI
de Lima et al. ^34^ (2021)	São Paulo	2011/2012	Prospective cohort	783	734	18.1	IADPSG adapted	24-39 weeks
Neto et al. ^53^ (2020)	Pernambuco	NI	Cross-sectional	NI	152	5.3	NI	NI
Nehab et al. ^54^ (2019)	Rio de Janeiro	March 2016/August 2017	Cross-sectional	NI	124	16.1	IADPSG adapted	Any
Ferreira et al. ^55^ (2020)	São Paulo	March 2015/March 2016	Retrospective cohort	229	151	13.9	NI	NI
Trujillo et al. ^8^ (2015) **	São Paulo, Rio de Janeiro, Rio Grande do Sul, Ceará, Bahia, Amazonas	May 1991/August 1995	Prospective cohort	5,564	4,926	18.0	IADPSG adapted	24-28 weeks
Barbieiri et al. ^56^ (2016)	São Paulo	March 2011/November 2012	Cross-sectional	1,446	799	19.0	ADA 2010	After 24 weeks
Renz et al. ^18^ (2015) ***	Rio Grande do Sul	September 2009/July 2012	Cross-sectional	283	262	15.3	IADPSG adapted	After 28 weeks
Rocha et al. ^57^ (2020)	Rio Grande do Sul	October 2016/December 2017	Prospective cohort	154	133	13.5	IADPSG adapted	NI
Peixoto et al. ^58^ (2016)	Minas Gerais	February 2012/March 2015	Retrospective cohort	1,740	817	8.6	IADPSG	24-28 weeks
Chume et al. ^59^ (2021)	Rio Grande do Sul	September 2009/July 2012	Cross-sectional	149	149	18.8	IADPSG adapted	24-28 weeks
Ayach et al. ^38^ (2010)	Mato Grosso do Sul	NI	Prospective cohort	341	279	4.3	Imprecise criteria	24-28 weeks
Possa & Oliveira ^60^ (2019)	Paraná	2016	Retrospective cohort	700	700	6.2	IADPSG adapted	24-28 weeks
Foratori-Junior et al. ^61^ (2021) ^#^	São Paulo	February 2019/November 2019	Prospective cohort	73	60	10.0	IADPSG adapted	32-36 weeks
Silva de Morais et al. ^62^ (2020)	Rio de Janeiro	September 2014/February 2017	Prospective cohort	243	214	14.9	NI	NI
Morais et al. ^63^ (2019)	Rio Grande do Sul	April/May 2017	Cross-sectional ^##^	28	20	5.0	NI	NI
Pereira et al. ^37^ (2017)	Rio Grande do Norte	2013	Cross-sectional ^##^	NI	200	1.6	NI	NI
Zapelini et al. ^64^ (2015) ^###^	Santa Catarina	August 2013/April 2014	Cross-sectional	NI	506	14.4	IADPSG	24-28 weeks
Oliveira et al. ^65^ (2015)	Alagoas	2013	Cross-sectional	NI	217	6.5	IADPSG adapted	NI
Alves et al. ^31^ (2014)	Bahia, Pernambuco	April 2011/January 2012	Cross-sectional ^##^	1,459	1,340	20.8	WHO 1999	NI
Nascimento et al. ^33^ (2016)	Pernambuco	November 2011/February 2014	Prospective cohort	974	841	10.8	IADPSG adapted	24-28 weeks

ADA: American Diabetes Association; GTDG: Brazilian Diabetes and Pregnancy Task Force; IADPSG: International Association of Diabetes in Pregnancy Study Group; NI: not informed; WHO: World Health Organization.

* This study also used the ADA 2010 diagnostic criteria, whose prevalence was 5.8%;

** Besides the IADPSG criteria, this study also used seven different adaptations from original IADPSG diagnostic criteria (prevalences of 2.7%; 12.7%; 15.6%; 17%; 3.1%; 2.7%; 4.5%, respectively), one from ADA 2010 (prevalence of 2.3%), one from WHO 1999 (prevalence of 7.1%) and two from WHO 1999 adapted (prevalence of 8% and 11.9%);

*** This study also used the WHO 1999 diagnostic criteria, whose prevalence was 27.48%;

^#^ The study did not present the gestational diabetes mellitus prevalence in the scientific article. This indicator was calculated based on the number of women with gestational diabetes mellitus and the population sample;

^##^ Descriptive prevalence study;

^###^ This study also used the ADA 2010 diagnostic criteria, whose prevalence was 0.6%, performed at any time up to the 34th week of pregnancy.

Gestational diabetes mellitus prevalence ranged from 1.6% to 40.2%. As for the gestational diabetes mellitus diagnostic criteria, 14 studies used the IADPSG adapted, five used the IADPSG, two the WHO 1999, two the ADA 2010, one the GTDG 2001, and one failed to specify the criterion used. Additionally, seven studies failed to report the criteria used for gestational diabetes mellitus diagnosis (Table 1).

Risk of bias analysis based on the instrument by Hoy et al. ^14^ found that five studies (15.6%) had a low risk of bias, 13 (37.5%) had a moderate risk of bias and 15 (46.8%) a high risk (Table 2). As 28 (82.4%) out of the 34 articles were characterized as moderate or high risk of bias, this indicates a vulnerability of the studies. The item with the highest frequency of “no” answers referred to the use of a representative sample (84.3%), whereas the item with the most “yes” answers concerns the use of the same diagnostic method for all evaluated pregnant women (96.9%). Only the study by Renz et al. ^18^ had all risk of bias criteria contemplated for avoidability, thus presenting the highest number of positive responses. Conversely, the study by Siqueira et al. ^19^ met none of the criteria, having the highest number of negative answers.

**Table 2:** Risk of bias assessment of studies included in the systematic review and meta-analysis of gestational diabetes mellitus prevalence in Brazil between 2010 and 2021.

Study (Year)	External validity					Internal validity					Total “yes” answers	Risk of bias summary *
	Representative sample?	Representative sampling frame?	Random selection or by census?	Response rate ≥ 75%?	Data collection through subjects?	Acceptable diagnostic criteria?	Reliable/ Validated test for gestational diabetes mellitus?	Same method used for all?	G estational diabetes mellitus test adequate for gestational age?	Appropriate prevalence calculation?		
Pinheiro et al. ^43^ (2018)	No	No	No	No	Yes	Yes	Yes	Yes	No	Yes	5	High
do Nascimento et al. ^44^ (2019)	No	No	No	No	Yes	Yes	Yes	Yes	Yes	Yes	6	Moderate
Nunes et al. ^45^ (2020)	No	Yes	No	No	No	Yes	Yes	Yes	Yes	No	5	High
dos Santos et al. ^46^ (2020)	No	Yes	No	No	No	Yes	Yes	Yes	No	Yes	5	High
Alves et al. ^47^ (2020)	No	No	No	Yes	Yes	Yes	Yes	Yes	Yes	Yes	7	Moderate
Nicolosi et al. ^48^ (2020)	No	No	No	No	Yes	Yes	Yes	Yes	No	Yes	5	High
Zhao et al. ^49^ (2016)	No	Yes	Yes	No	Yes	Yes	Yes	Yes	Yes	Yes	8	Low
Guttier et al. ^32^ (2019)	Yes	Yes	Yes	Yes	Yes	No	No	Yes	No	Yes	7	Moderate
Sirimarco et al. ^50^ (2017)	No	Yes	No	Yes	No	Yes	Yes	Yes	No	Yes	6	Moderate
Santos et al. ^51^ (2012)	No	No	No	Yes	Yes	Yes	Yes	Yes	No	Yes	6	Moderate
Fagundes et al. ^52^ (2016)	No	No	No	Yes	Yes	Yes	Yes	Yes	Yes	Yes	7	Moderate
Siqueira et al. ^19^ (2019)	No	No	No	No	No	No	No	No	No	No	0	High
de Lima et al. ^34^ (2021)	No	No	No	Yes	Yes	Yes	Yes	Yes	Yes	Yes	7	Moderate
Neto et al. ^53^ (2020)	No	Yes	Yes	No	Yes	No	No	Yes	No	Yes	5	High
Nehab et al. ^54^ (2019)	No	No	No	No	Yes	Yes	Yes	Yes	No	Yes	5	High
Ferreira et al. ^55^ (2020)	No	No	No	No	No	No	No	Yes	No	No	1	High
Trujillo et al. ^8^ (2015)	No	No	No	Yes	Yes	Yes	Yes	Yes	No	No	5	High
Barbieiri et al. ^56^ (2016)	No	No	No	No	Yes	Yes	Yes	Yes	No	Yes	5	High
Renz et al. ^18^ (2015)	Yes	Yes	Yes	Yes	Yes	Yes	Yes	Yes	Yes	Yes	10	Low
Rocha et al. ^57^ (2020)	Yes	Yes	Yes	Yes	No	Yes	Yes	Yes	No	Yes	8	Low
Peixoto et al. ^58^ (2016)	No	No	No	No	No	Yes	Yes	Yes	Yes	Yes	5	High
Chume et al. ^59^ (2021)	No	No	No	Yes	Yes	Yes	Yes	Yes	Yes	Yes	7	Moderate
Ayach et al. ^38^ (2010)	No	No	No	Yes	Yes	Yes	Yes	Yes	Yes	Yes	7	Moderate
Possa & Oliveira ^60^ (2019)	No	No	No	Yes	Yes	Yes	Yes	Yes	Yes	Yes	7	Moderate
Foratori-Junior et al. ^61^ (2021)	No	No	No	Yes	No	Yes	Yes	Yes	No	Yes	5	High
Silva de Morais et al. ^62^ (2020)	No	No	No	Yes	No	No	No	Yes	No	Yes	3	High
Morais et al. ^63^ (2019)	No	Yes	No	No	Yes	No	No	Yes	No	Yes	4	High
Pereira et al. ^37^ (2017)	No	Yes	Yes	No	Yes	No	No	Yes	No	Yes	5	High
Zapelini et al. ^64^ (2015)	No	Yes	No	No	No	Yes	Yes	Yes	Yes	Yes	6	Moderate
Oliveira et al. ^65^ (2015)	Yes	Yes	No	No	Yes	Yes	Yes	Yes	Yes	Yes	8	Low
Alves et al. ^31^ (2014)	Yes	Yes	No	Yes	Yes	Yes	Yes	Yes	Yes	Yes	9	Low
Nascimento et al. ^33^ (2016)	No	No	No	Yes	Yes	Yes	Yes	Yes	Yes	No	6	Moderate

* Risk of bias rating: low risk of bias (8 or more “yes” answers); moderate (with 6 to 7 “yes” answers); high (with 5 or less “yes” answers).

Overall certainty of evidence rating was low. Quality assessment showed weaknesses in inconsistency and indirectness (Box 1).

**Box 1:** GRADE (Grades of Recommendation, Assessment, Development, and Evaluation) assessment of papers on gestational diabetes mellitus prevalence in Brazil between 2010 and 2021.

QUALITY ASSESSMENT							
Studies	Study design	Risk of bias	Inconsistency	Imprecision	Indirectness	Publication bias	Other considerations
32	Observational studies	Not serious *	Serious **	Not serious ***	Serious ^#^	Not serious ^##^	NA
Prevalence (95%CI): 0.14 (0.11; 0.16)				Quality: Low ⊕⊕○○				

95%CI: 95% confidence interval; NA: not applicable.

* Although most studies were classified as high risk of bias according to Hoy et al. ^14^, the sensitivity analysis revealed minimal disparity in the gestational diabetes mellitus prevalence between studies with low risk (0.12) and high risk (0.14) ratings. Thus, we deduced that the limitations of the weaker studies did not importantly bias the results. Consequently, we did not rate down the confidence rating;

** Considerable heterogeneity in results across studies was observed, ranging from 0.03 to 0.40. Consequently, we opted to downgrade the confidence level;

*** Besides the high heterogeneity among studies, the inclusion of a large number of studies in this systematic review resulted in a narrow 95%CI range for prevalence (0.11; 0.16). We decided to not rate down the confidence rating;

^#^ Noticeable heterogeneity was observed in study characteristics, such as variations in diagnostic criteria, gestational age of the populations studied, and sample sizes. According to Hoy et al. ^14^ instrument, a large number of studies presented limitations in criteria related to external validity. We opted to downgrade the confidence level;

^##^ Publication bias was verified using Egger’s test (p = 0.003), as well as the funnel plot. However, the gestational diabetes mellitus pooled prevalence (14%) is included in the 95%CI of the trim-and-fill prevalence estimation (8.8; 15.9). Consequently, we decided to not rate down the confidence rating.

**Figure 2:**
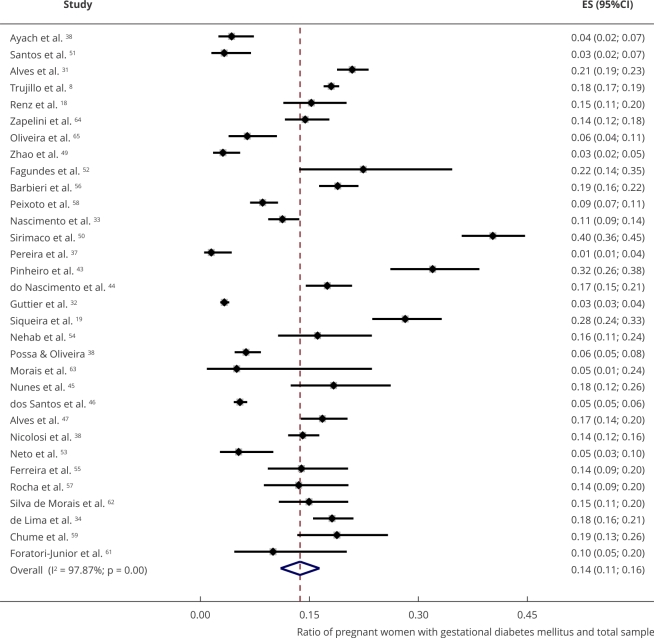
Gestational diabetes mellitus prevalence forest plot of studies published in Brazil between 2010 and 2021.

**Figure 3:**
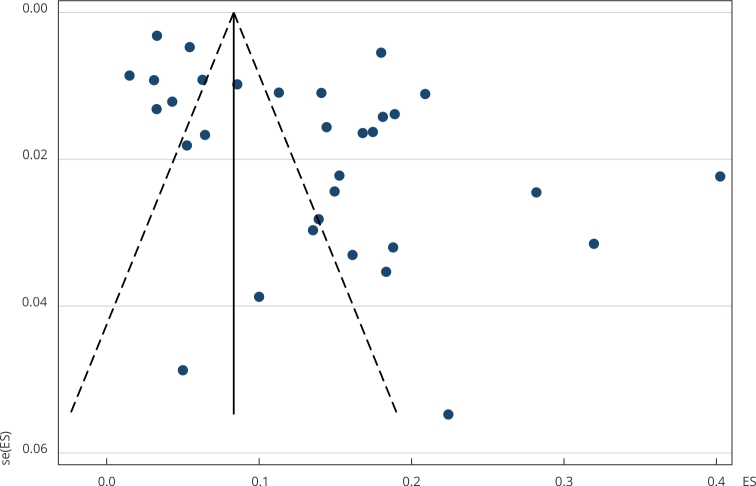
Funnel plot with pseudo 95% confidence intervals on the ratio of pregnant women with gestational diabetes mellitus in Brazil between 2010-2021.

The meta-analysis included all 32 articles to estimate the gestational diabetes mellitus pooled prevalence of 14% (95%CI: 11.0; 16.0) with a heterogeneity between studies (I^2^) of 97.9% (p < 0.001) (Figure 2). Regarding publication bias, the Egger’s test with a p-value of 0.003 and the funnel plot indicate its presence (Figure 3). To estimate the potential impact of publication bias on the gestational diabetes mellitus pooled prevalence, we performed a trim-and-fill test imputing two studies on the left side of the funnel plot, resulting in a pooled prevalence of 12.3% (95%CI: 8.8; 15.9).

95%CI: 95% confidence interval; ES: effect size.

ES: effect size; se(ES): standard error of the effect size.

Other meta-analyses stratified by some characteristics were conducted to analyze their influence on the gestational diabetes mellitus pooled prevalence (Table 3) (forest plots are presented in Supplementary Material - Figures S1, S2 and S3; https://cadernos.ensp.fiocruz.br/static//arquivo/suppl-e00064919_9189.pdf). Risk of bias analysis classified five (15.6%) studies as low risk of bias, which analyzed 1,645 individuals and 12% (95%CI: 3.0; 20.0) pooled prevalence; 12 articles (37.5%) as moderate risk of bias, totaling 7,515 women and 14% (95%CI: 10.0; 19.0) pooled prevalence, and 15 studies as high risk of bias, with 14% of gestational diabetes mellitus pooled prevalence (95%CI: 10.0; 18.0) and a population of 11,460 participants.

**Table 3:** Meta-analysis stratified by risk of bias, diagnostic criteria and country region with study data concerning gestational diabetes mellitus pooled prevalence in Brazil between 2010 and 2021.

Subgroup	Studies	Full sample	Prevalence (95%CI)	Heterogeneity		
				Q value	I^2^	p-value *
Risk of bias						0.870
High	15	11,460	0.14 (0.10; 0.18)	584.33	97.60	
Moderate	12	7,515	0.14 (0.10; 0.19)	557.04	98.03	
Low	5	1,645	0.12 (0.03; 0.20)	163.34	97.55	
Diagnostic criteria						0.450
IADPSG	5	2,862	0.15 (0.10; 0.20)	32.74	87.78	
IADPSG adapted	14	9,803	0.14 (0.11; 0.18)	437.36	97.03	
Other criteria **	6	3,699	0.15 (0.06; 0.24)	421.41	98.81	
Not informed	7	4,256	0.10 (0.05; 0.15)	143.20	95.81	
Country region						0.170
Northeast	7	3,754	0.11 (0.05; 0.18)	244.71	97.55	
Central-West	2	780	0.09 (0.07; 0.11)	-	-	
Southeast	9	1,991	0.14 (0.09; 0.18)	146.04	94.52	
South	10	8,653	0.13 (0.09; 0.16)	203.66	95.58	

95%CI: 95% confidence interval; ADA: American Diabetes Association; GTDG: Brazilian Diabetes and Pregnancy Task Force; IADPSG: International Association of Diabetes in Pregnancy Study Group; WHO: World Health Organization.

* p-value related to the difference between subgroups;

** The other diagnostic criteria for gestational diabetes mellitus used were the ADA 2010 (5 studies), WHO 1999 (4 studies), WHO 1999 adapted (1 study), GTDG 2001 (1 study) and unspecified criteria (1 study).

As for diagnostic criteria, the gestational diabetes mellitus pooled prevalence was 15% (95%CI: 10.0; 20.0), 14% (95%CI: 11.0; 18.0), 15% (95%CI: 6.0; 24.0) and 10% (95%CI: 5.0; 15.0), respectively, for the IADPSG, IADPSG adapted, other criteria and unspecified criteria. Analysis by country region showed that most studies were conducted in the Southeast and South regions, with 14% (95%CI: 0.09; 0.18) and 13% (95%CI: 9.0; 16.0) gestational diabetes mellitus pooled prevalences, respectively. Northeast presented a pooled prevalence of 11% (95%CI: 5.0-18.0) and the Central-West, 9% (95%CI: 7.0; 11.0).

**Figure 4:**
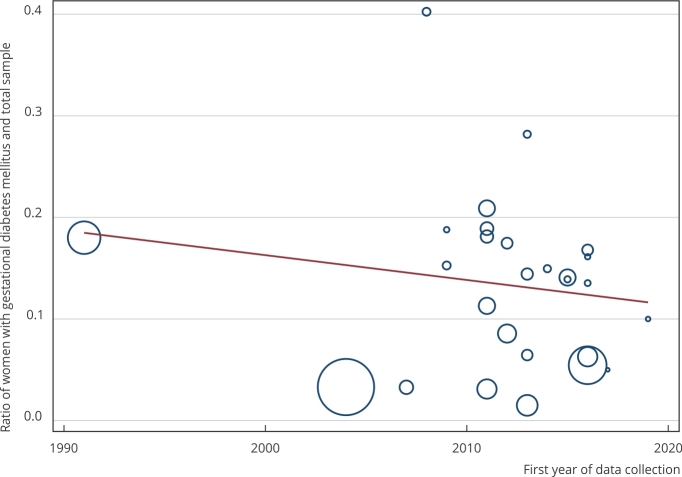
Meta-regression analysis on the ratio of pregnant women with gestational diabetes mellitus and the year of data collection in the studies published in Brazil between 2010-2021.

Finally, the results of the meta-regression analysis for random effects (Figure 4) showed that the variable “year of data collection” did not significantly contribute to heterogeneity, presenting a coefficient equal to -0.002 (95%CI: -0.009; 0.004) and a p-value of 0.439.

## Discussion

This systematic review and meta-analysis estimated the gestational diabetes mellitus pooled prevalence in Brazil at 14% (95%CI: 11.0; 16.0) from analyzing 32 studies, totaling a sample of 21,942 pregnant women. Moreover, it assessed the pooled prevalence according to country region, the diagnostic criteria used and the risk of bias.

Brazil’s estimated gestational diabetes mellitus pooled prevalence is similar to that is found in mainland China (14.8%; 95%CI: 12.8; 16.7) ^20^, Australia (14%) ^21^ and Africa (13.6%; 95%CI: 10.99; 16.23) ^10^. Pooled prevalence was 11.7% (95%CI: 10.7; 12.6) in Eastern Mediterranean ^15^ and 11.5% (95%CI: 10.9; 12.1) in Asia ^3^, specifically reaching 10.07% (95%CI: 6.47; 15.68) in East and Southeast Asia ^22^. In Europe, the value was 10.9% (95%CI: 10; 11.8) ^23^; 8.2% (95%CI: 7.5; 8.9) in the United States ^24^; 7.7% (95%CI: 1.9; 27.9) in Turkey ^25^; 3.4% (95%CI: 18.6; 1.3) in Iran ^26^; and 2.3% in Japan ^27^.

According to the *Diabetes Atlas* of the International Diabetes Federation (IDF) ^7^, in 2021 the estimated gestational diabetes mellitus prevalence in South and Central America was 10.4% (95%CI: 10.1; 10.7), below the pooled prevalence found in this systematic review. A study conducted in Chile found an even lower prevalence, 7.6% (95%CI: 7.5; 7.8) ^28^. Studies in countries like Argentina and Peru, in turn, observed a higher prevalence than those found in Brazil, 24.9% and 16%, respectively ^29^ ^,^ ^30^.

A Brazilian study conducted with greater robustness (larger sample and low risk of bias) showed a prevalence of 20.8% ^31^. Other studies with comparatively larger samples, but with a moderate risk of bias, presented greater variability, probably due to the sources of heterogeneity discussed later: 3.3% ^32^, 10.8% ^33^ and 18,1% ^34^.

Importantly, this was the first systematic review and meta-analysis on gestational diabetes mellitus in the country. Previously, based on a cohort study ^35^ from 1991 to 1995, the 2006 Brazilian guidelines ^36^ cited a prevalence between only 2.4% (95%CI: 2.0; 2.9) and 7.2% (95%CI: 6.5; 7.9) according to 2000 ADA and 1999 WHO criteria, respectively. Conversely, the 2017 Brazilian consensus ^1^ points to a prevalence of around 18% (95%CI: 16.9; 19.0) based on a cohort study ^8^ using IADPSG criteria. Our study presents a considerably higher estimate than older investigations founded on previous diagnostic criteria and a similar, but lower, estimate to a newer research using the updated criteria.

Regarding pooled prevalences analyzed by region, the Northeast showed a pooled prevalence of 11%, due to the disparity between the studies with 20.8% ^30^ and 1.6% ^37^ values, and the Central-West of 9%, given the 28.2% ^18^ and 4.3% ^38^ values. A possible explanation for data variability is the asymmetry in socioeconomic conditions and access to health services between Brazilian regions, influencing the number of diagnoses. Screening is made difficult by factors such as housing conditions, family income, schooling level, urbanization, water supply and sanitation thus increasing the chances of complications during pregnancy ^37^. This hypotheses aligns with in a study conducted in India ^39^, which pointed to a considerable variation in gestational diabetes mellitus prevalence by state, socioeconomic level and demographic factors, as well as the correlation of areas with few economic resources allocated to gestational diabetes mellitus screening with lower prevalence levels. Hence, socioeconomic and care factors may influence this decrease in regional prevalence.

As for the diagnostic criteria used, we observed a weakness in the studies homogeneity. A total of five different diagnostic criteria were identified in the analyzed articles, in addition to those lacking this information. IADPSG (five studies) and the adapted IADPSG (14 studies) were the most used, frequently performing the OGTT 75g in a period different from that established in the original instrument. The stratified meta-analysis found a higher pooled prevalence (15%; 95%CI: 10.0; 20.0) in articles that employed the original criterion and a lower pooled prevalence (14%; 95%CI: 11.0; 18.0), in those with some adaptation, showing a possible decrease in diagnostic sensitivity. Similarly, when comparing the IADPSG criterion with the 2010 ADA, other studies have found higher diagnostic rates with the former ^40^ ^,^ ^41^ ^,^ ^42^, confirming its greater sensitivity.

Regarding risk of bias, although most of the articles (82.4%) analyzed presented moderate or high risk, proportional values were obtained among low, moderate and high risk. A gestational diabetes mellitus pooled prevalence of 12% was found among low-risk studies; of 14% among moderate-risk studies, and of 14% among high-risk studies. Representative sample (27 studies) and random or census selection (26 studies) were the most frequent risks of bias, whereas using different diagnostic methods for all participants occurred only once. Despite the importance of study quality for selecting the best evidence, the risk of bias was not a factor of great influence on distorting the results found.

Study limitations include the disparity in the number of studies from different regions and the lack of detailed information about methodology and gestational diabetes mellitus measurement criteria in some articles. Thus, threshold value changes in identifying gestational diabetes mellitus would inevitably cause high heterogeneity in the results. Additionally, the meta-analysis included studies with small sample sizes, which may result in data with high analytical variability, and different designs (whether prospective or retrospective cohort, cross-sectional study, diagnostic or descriptive test).

Despite achieving the main study objective, we did not evaluate the factors that may influence gestational diabetes mellitus prevalence. Most studies have not evaluated the gestational diabetes mellitus effects on maternal and fetal outcomes and were conducted in Southeastern and Southern municipalities, causing a great risk of bias in data interpretation and generalization for other locations which were not included in the meta-analysis or had few articles analyzed in comparison. Similarly, none of the studies included in this systematic review used a national population base pointing to the need for new nationally representative research.

Despite these limitations, this is the first meta-analysis conducted in Brazil about gestational diabetes mellitus prevalence stratified by region and with analysis of risk of bias and methodological quality of the publications, helping with data interpretation.

## Conclusion

This study provided evidence on estimated gestational diabetes mellitus occurrence in Brazil between 2010 and 2021. Data summarized in the meta-analysis showed a gestational diabetes mellitus pooled prevalence of 14%. Country region, the diagnostic criteria used and study quality influenced the resulting pooled prevalence indicator. However, the high heterogeneity between the studies hindered to summarize the findings.

To the best of our knowledge, this meta-analysis is the first to provide evidence on the national gestational diabetes mellitus pooled prevalence, a key factor in understanding and characterizing the epidemiology of the condition. Given the evidence generated, the issue may trigger greater interest in health managers to address the disease. The current national scenario requires planning to manage the condition. Screening and diagnosis, based on standardized criteria, as well as preventive actions for gestational diabetes mellitus control and adequate patient management could potentially reduce this disease’s burden.
