# Supplementary material for: Gestational diabetes mellitus prevalence in Brazil: a systematic review and meta-analysis
Source: Cad Saude Publica. 2024 Sep 9;40(8):e00064919. doi: 10.1590/0102-311XEN064919 (PMC11386532; doi:10.1590/0102-311XEN064919)
Supplement: Supplementary file 1 [file 1678-4464-csp-40-08-EN064919-s.pdf]

SUPPLEMENTARY MATERIAL

Box S1 Search terms used for final research about prevalence of gestational diabetes mellitus in women pregnant in Brazil between 2010-2021.

DATA BASED USED	SEARCH TERM	ITEMS FOUND	DATE
PubMed	(diabetes OR hyperglycemia OR "glucose intolerance" OR "gestational diabetes" OR "impaired glucose tolerance" OR "impaired fasting glucose" OR "diabetes mellitus" OR "postprandial glucose tolerance" OR "glucose tolerance") AND (pregnan* OR gestation* OR Gravid* OR gestational diabetes) AND (Brazil OR Acre OR Alagoas OR Amapá OR Amazonas OR Bahia OR Ceará OR Distrito Federal OR Espírito Santo OR Goiás OR Maranhão OR Mato Grosso OR Mato Grosso do Sul OR Minas Gerais OR Pará OR Paraíba OR Paraná OR Pernambuco OR Piauí OR Rio de Janeiro OR Rio Grande do Norte OR Rio Grande do Sul OR Rondônia OR Roraima OR Santa Catarina OR São Paulo OR Sergipe OR Tocantins)	871	06/Sep/2021
Scopus	(diabetes OR hyperglycemia OR "glucose intolerance" OR "gestational diabetes" OR "impaired glucose tolerance" OR "impaired fasting glucose" OR "diabetes mellitus" OR "postprandial glucose tolerance" OR "glucose tolerance") AND (pregnan* OR gestation* OR Gravid* OR gestational diabetes) AND (Brazil OR Acre OR Alagoas OR Amapá OR Amazonas OR Bahia OR Ceará OR Distrito Federal OR Espírito Santo OR Goiás OR Maranhão OR Mato Grosso OR Mato Grosso do Sul OR Minas Gerais OR Pará OR Paraíba OR Paraná OR Pernambuco OR Piauí OR Rio de Janeiro OR Rio Grande do Norte OR Rio Grande do Sul OR Rondônia OR Roraima OR Santa Catarina OR São Paulo OR Sergipe OR Tocantins)	23	06/Sep/2021
SciELO	(diabetes mellitus gestacional) OR (diabetes) OR (hiperglicemia) OR (Intolerância à Glucose) OR (diabetes gestacional) OR (Intolerância à Glicose Diminuída) OR (diabetes mellitus) AND (gravid*) OR (gesta*) AND (Rio Grande do Sul) OR (Santa Catariana) OR (Paraná) OR (São Paulo) OR (Rio de Janeiro) OR (Minas Gerais) OR (Espírito Santo) OR (Mato Grosso do Sul) OR (Goiás) OR (Mato Grosso) OR (Distrito Federal) OR (Bahia) OR (Sergipe) OR (Alagoas) OR (Pernambuco) OR (Paraíba) OR (Rio Grande do Norte) OR (Ceará) OR (Maranhão) OR (Piauí) OR (Tocantins) OR (Pará) OR (Amazonas) OR (Rondônia) OR (Acre) OR (Bra?il)	266	06/Sep/2021
VHL	(Gestational diabetes mellitus) OR (diabetes) OR (hyperglycemia) OR (glucose intolerance) OR (gestational diabetes) OR (impaired glucose tolerance) OR (impaired fasting glucose) OR (diabetes mellitus) OR (postprandial glucose tolerance) OR (glucose tolerance) AND (Bra?il)	197	06/Sep/2021
LILACS	(Gestational diabetes mellitus) OR (diabetes) OR (hyperglycemia) OR (glucose intolerance) OR (gestational diabetes) OR (impaired glucose tolerance) OR (impaired fasting glucose) OR (diabetes mellitus) OR (postprandial glucose tolerance) OR (glucose tolerance) AND (Bra?il)	1.764	12/Oct/2021

Figure S1 Gestational diabetes mellitus prevalence forest plot of studies analyzed in Brazil between 2010 and 2021 stratified by risk of bias.

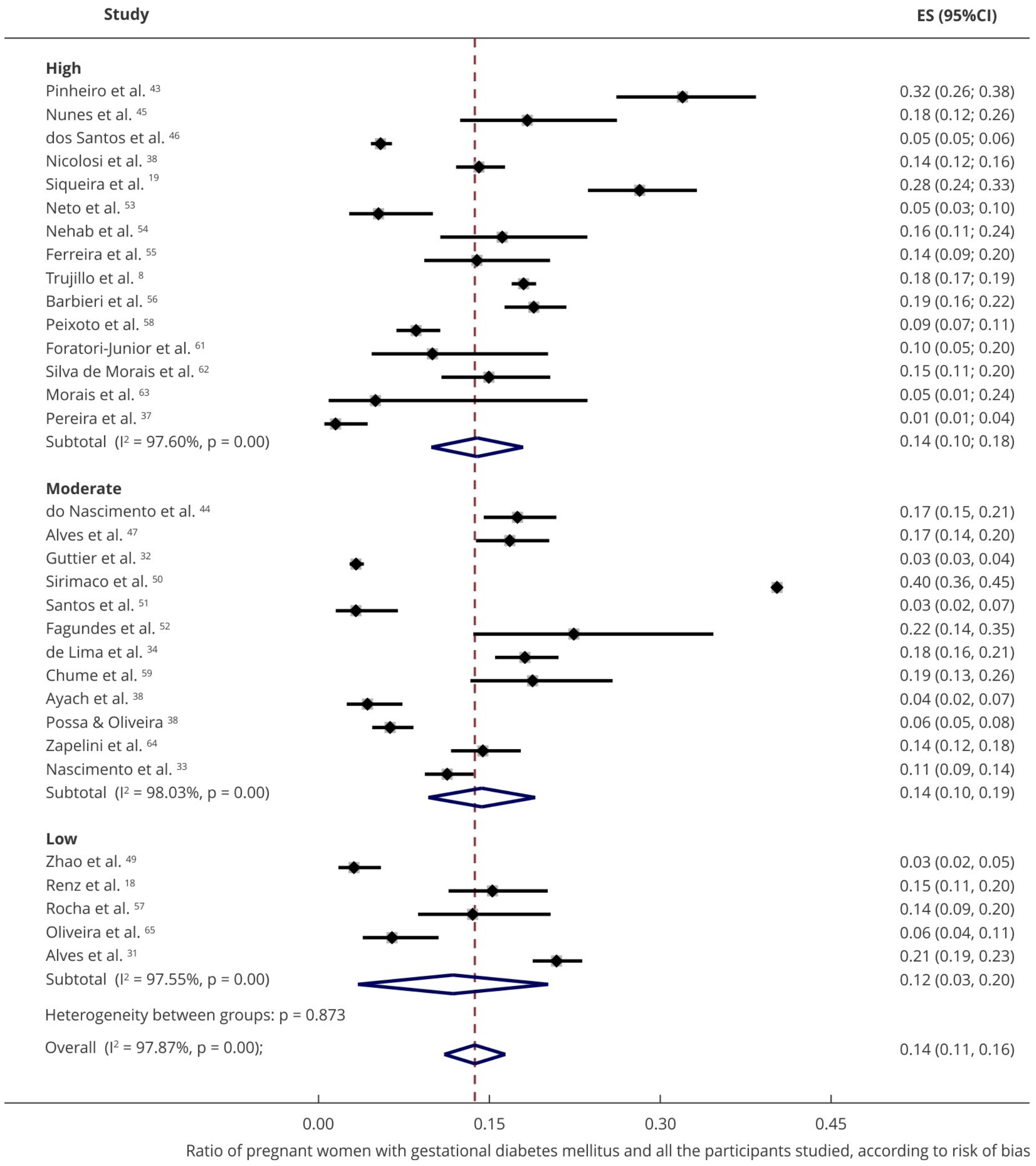

95%CI: 95% confidence interval; ES: effect size.

Figure S2 Gestational diabetes mellitus prevalence forest plot of studies analyzed in Brazil between 2010 and 2021 stratified by diagnostic criteria.

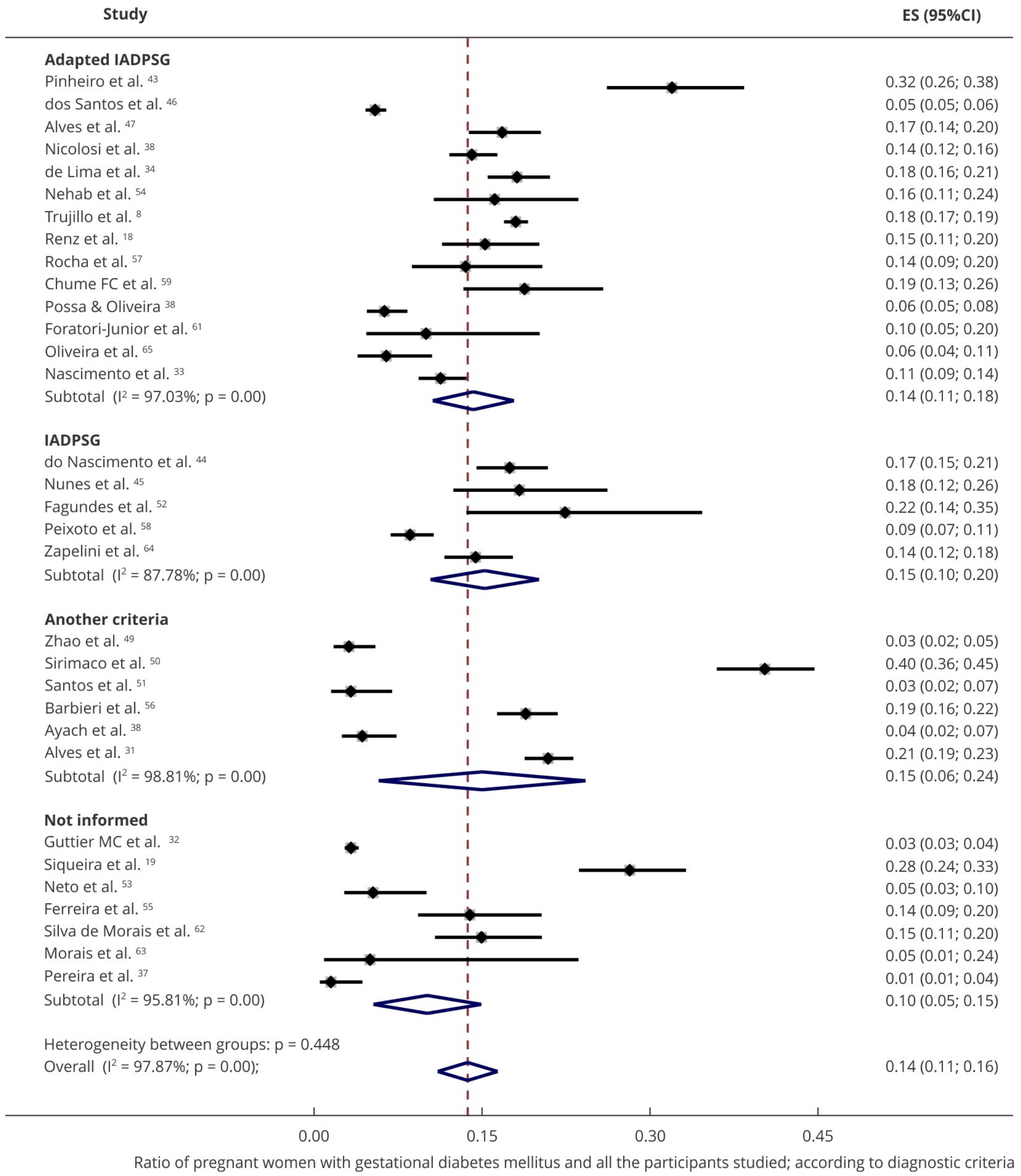

95%CI: 95% confidence interval; ES: effect size.

Figure S3 Gestational diabetes mellitus prevalence forest plot of studies analyzed in Brazil between 2010 and 2021 stratified by country region.

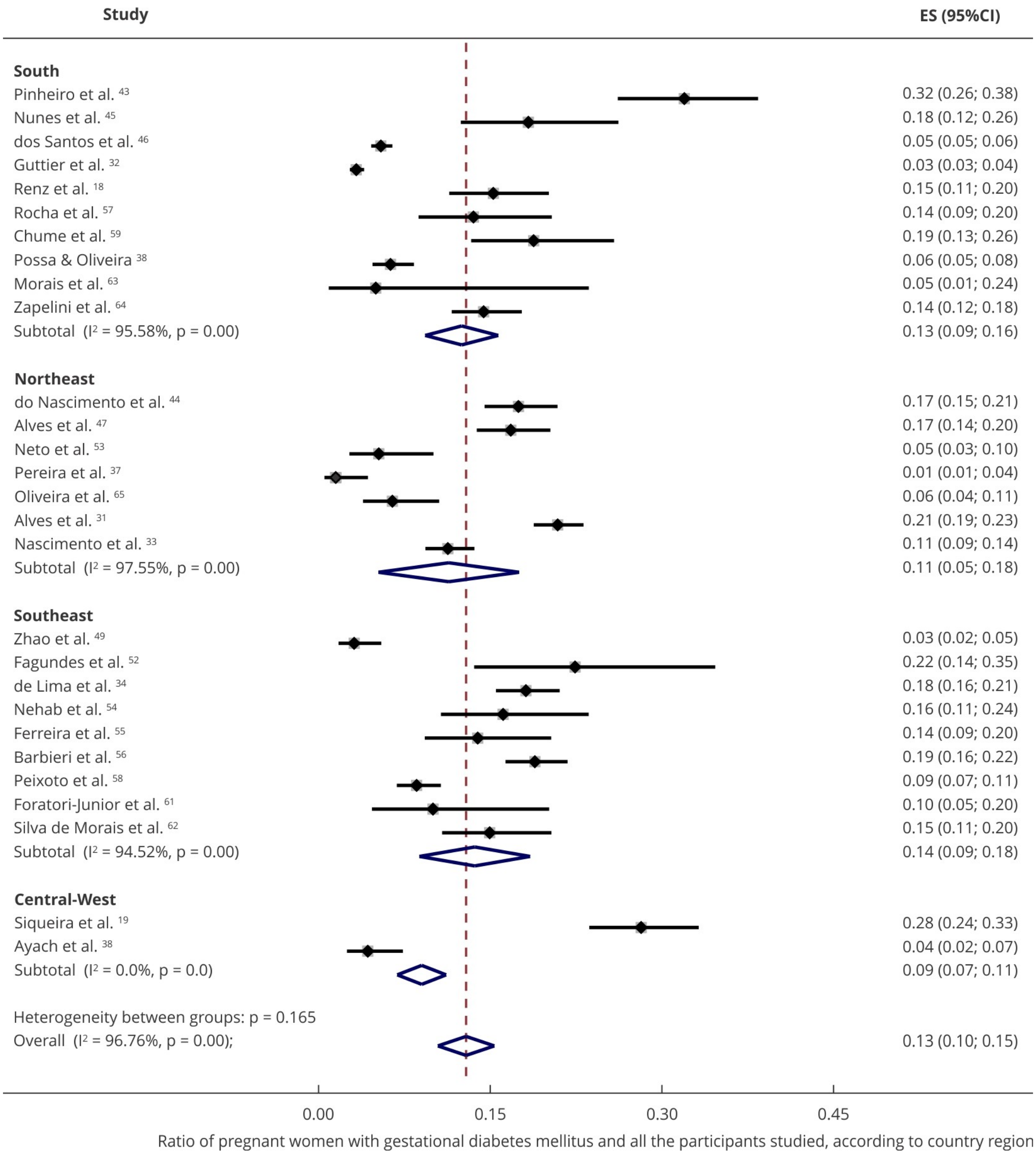

95%CI: 95% confidence interval; ES: effect size.
